# Expression and functional role of CRIPTO-1 in cutaneous melanoma

**DOI:** 10.1038/bjc.2011.324

**Published:** 2011-08-23

**Authors:** A De Luca, L Lamura, L Strizzi, C Roma, A D'Antonio, N Margaryan, G Pirozzi, M-Y Hsu, G Botti, E Mari, M J C Hendrix, D S Salomon, N Normanno

**Affiliations:** 1 Cell Biology and Biotherapy Unit, Research Department, INT-Fondazione Pascale, Naples 80131, Italy; 2 Children's Memorial Research Center, Robert H. Lurie Comprehensive Cancer Center, Northwestern University Feinberg School of Medicine, Chicago, IL 60614-4314, USA; 3 Laboratory of Pharmacogenomic, Centro di Ricerche Oncologiche di Mercogliano–CROM, Mercogliano (AV) 83013, Italy; 4 Pathology Unit, S. Giovanni di Dio e Ruggi d’Aragona Hospital, Salerno 84100, Italy; 5 Department of Dermatology, Boston University School of Medicine, Boston, MA 02118, USA; 6 Pathology Unit, INT-Fondazione Pascale, Naples 80131, Italy; 7 AstraZeneca, Basiglio (MI) 20080, Italy; 8 Mammary Biology and Tumorigenesis Laboratory, Center for Cancer Research, National Cancer Institute, National Institutes of Health, Bethesda, MD 20892, USA

**Keywords:** CRIPTO-1, melanoma, invasion, therapy, saracatinib

## Abstract

**Background:**

CRIPTO-1 (CR-1) is involved in the pathogenesis and progression of human carcinoma of different histological origin. In this study we addressed the expression and the functional role of CR-1 in cutaneous melanoma.

**Methods:**

Expression of CR-1 protein in melanomas and melanoma cell lines was assessed by immunohistochemistry, western blotting and/or flow cytometry. Levels of mRNA were evaluated by real-time PCR. Invasion assays were performed in Matrigel-coated modified Boyden chambers.

**Results:**

Expression of CR-1 protein and/or mRNA was found in 16 out of 37 primary human cutaneous melanomas and in 12 out of 21 melanoma cell lines. Recombinant CR-1 protein activated in melanoma cells c-Src and, at lesser extent, Smad signalling. In addition, CR-1 significantly increased the invasive ability of melanoma cells that was prevented by treatment with either the ALK4 inhibitor SB-431542 or the c-Src inhibitor saracatinib (AZD0530). Anti-CR-1 siRNAs produced a significant inhibition of the growth and the invasive ability of melanoma cells. Finally, a close correlation was found in melanoma cells between the levels of expression of CR-1 and the effects of saracatinib on cell growth.

**Conclusion:**

These data indicate that a significant fraction of cutaneous melanoma expresses CR-1 and that this growth factor is involved in the invasion and proliferation of melanoma cells.

Cutaneous melanoma is an aggressive disease that arises from melanocytes, specialised pigmented cells that are found predominantly in the basal layer of the skin (Gray-Schopfer *et al*, 2007). The melanoma progression is characterised by a step-wise process that generally encompasses the following: common acquired nevus; dysplastic nevus; a radial-growth phase, the first recognisable malignant step in which the cells proliferate predominantly intraepidermally; a vertical-growth phase in which the cells proliferate extensively in the dermis and acquire metastatic potential; and metastatic melanoma, when tumour cells have spread to distant tissues (Miller and Mihm, 2006). Metastatic melanoma is refractory to current therapies and has a very poor prognosis, with a 5-years survival rate of 5–14% (Miller and Mihm, 2006; Gray-Schopfer *et al*, 2007).

Human CRIPTO-1 (CR-1), also defined as teratocarcinoma-derived growth factor 1 (TDGF1), is a cell membrane protein that belongs to the epidermal growth factor (EGF)-CFC family (Bianco *et al*, 2005; Strizzi *et al*, 2005). This family of proteins is characterised by the presence of an N-terminal signal peptide, a modified EGF-like domain, a cysteine-rich CFC motif and a glycosylphosphatidylinositol-linkage signal at the C-terminus (Strizzi *et al*, 2005).

CRIPTO-1 functions through at least three different signalling pathways: (1) as a co-receptor for the transforming growth factor beta (TGF-*β*)-related proteins Nodal and growth and differentiation factors 1 and 3; (2) as a ligand for glypican-1/c-Src/MAPK/PI3K-Akt signalling; and (3) as an inhibitor for Activin/TGF-*β* signalling (Bianco *et al*, 2005; Strizzi *et al*, 2005; Gray *et al*, 2006).

CRIPTO-1 has been shown to have an important role in vertebrate development (Bianco *et al*, 2005; Strizzi *et al*, 2005). Evidence suggests that CR-1 is also involved in the pathogenesis and progression of human carcinoma. CR-1 is not usually expressed in adult normal tissues, whereas high expression of CR-1 transcript and/or protein has been observed in different human carcinomas, including breast, colon, stomach, pancreas, lung, ovary, cervix and testis, and in leiomyosarcomas of the uterus (Normanno *et al*, 2001; Bianco *et al*, 2005; Strizzi *et al*, 2007). Overexpression of CR-1 can lead to *in vitro* transformation of human mammary epithelial cells and can promote mammary tumourigenesis in transgenic mice (Ciardiello *et al*, 1991; Sun *et al*, 2005; Wechselberger *et al*, 2005). In addition, overexpression of CR-1 increases the invasiveness and the resistance to anoikis of human breast cancer cells (Normanno *et al*, 2004a).

The high frequency of expression of CR-1 in different carcinoma types and the low expression in normal tissues suggest that this protein might be a suitable target for cancer therapy. Indeed, it has been observed that treatment with antisense oligonucleotides directed against CR-1 results in a significant inhibition of the *in vitro* and *in vivo* growth of human carcinoma cells of different histological origin, in which CR-1 can function as autocrine or paracrine growth factor (Ciardiello *et al*, 1994; De Luca *et al*, 1999, 2000; Normanno *et al*, 2004b). In addition, CR-1 might represent a suitable target for immunotherapy, considering its unique tissue distribution (Xing *et al*, 2004). In this regard, Renard *et al* have filed a patent (WO2008040759) proposing a vaccine for targeting CR-1 in cancer.

Expression of Nodal, an embryonic growth factor for which CR-1 acts as a co-receptor, is positively correlated with melanoma invasiveness and aggressiveness (Topczewska *et al*, 2006). Although preliminary findings from our group have shown that a subset of melanoma cells also express CR-1 on the cell membrane, the precise role of CR-1 in cutaneous melanoma remains to be determined (Strizzi *et al*, 2008, 2009).

In this study, we addressed the expression and the functional role of CR-1 in cutaneous melanoma. For this purpose, we assessed the expression of CR-1 in human melanoma biopsy samples and melanoma cell lines, and determined the effects of CR-1 signalling on the growth and invasiveness of human melanoma cells.

## Materials and methods

### Materials

The c-Src inhibitor Saracatinib (AZD0530) was kindly provided by AstraZeneca (Macclesfield, UK). The ALK4/5/7 inhibitor SB-431542 was purchased from Sigma (Milan, Italy). The recombinant human CR-1 and Nodal proteins were purchased from R&D Systems (Minneapolis, MN, USA).

### Immunohistochemistry

Immunohistochemical analysis for CR-1 expression was performed in paraffin-embedded, formalin-fixed tissues from 37 patients with primary cutaneous melanoma by using a specific anti-CR-1 antibody (Rockland Labs, Gilbertsville, PA, USA) as previously described (D’Antonio *et al*, 2002). Sections were obtained from the tissue archives of the INT-Fondazione Pascale, Naples, Italy and from Dr Mei-Yu Su, Boston MA, under Institutional Review Boards-approved protocols. The following scoring system was used: 1+, weak staining; 2+, moderate staining; 3+, intense staining. The specimens were judged positive when the intensity of staining was 2+ or 3+, and at least 10% of the tumour cells were stained.

### Cell culture

The following human melanoma cell lines were maintained in culture as previously described: M14, ROS 184, TRAR 60, LIMA, MAVI 74, CON 242, COPA 159, FORMICA, PLF 1, PLF 2, JR1, JR8, SBcl2, SBcl1, CIR 229, CHM-A, PES 47, PES 43, PES 41, ANAD 63 and COLO 36. Cell lines were from American Type Culture Collection (ATCC, Manassas, VA, USA) or have been established in the laboratories of the INT-Fondazione Pascale from human primary melanomas (Verschraegen *et al*, 1991; Leonetti *et al*, 1996; Pirozzi *et al*, 2000; Scala *et al*, 2006).

### Invasion assay

Invasion assays were performed in Matrigel-coated modified Boyden chambers (Cell Invasion Assay Kit, Chemicon/Millipore, Milan, Italy). Cell lines CON 242, COPA 159 and ROS 184 were cultured in medium without serum for 24 h. Then, serum-starved cells were trypsinised and added in the upper chamber (3 × 10^5^ cells per insert for CON 242, 2 × 10^5^ cells per insert for COPA 159, 2 × 10^5^ cells per insert for ROS 184). The upper chambers were treated with recombinant CR-1 protein (200 ng ml^−1^) alone or in combination with saracatinib (0.5 *μ*M) or SB-431542 (10 *μ*M). Iscove's modified Dulbecco's medium supplemented with 2% fetal bovine serum was used as a chemoattractant in the lower Boyden chamber. The Boyden chambers were incubated for 20 h at 37 °C. Non-invading cells were removed and the invading cells were stained with crystal violet stain solution (Chemicon). The solution was eluted with 10% acetic acid extraction buffer (Chemicon), transferred to wells of a 96-multiwell plate, and the absorbance was read at 595 nm in each well.

### Anchorage-dependent growth assay

The following cells were seeded in 96-well microtiter plates in serum-containing medium: CON 242 (15 × 10^3^ cells per well), COPA 159 (10 × 10^3^ cells per well), ROS 184 (12 × 10^3^ cells per well), SBcl1 (10 × 10^3^ cells per well) and SBcl2 (10 × 10^3^ cells per well). After 24 h, the medium was replaced and cells were treated with the indicated concentrations of saracatinib. After 72 h, the tetrazolium-based (MTT) colorimetric assay was performed to determine the sensitivity of the cell lines to saracatinib, as previously described (Normanno *et al*, 2006).

### Transfection with siRNA

The following cells were cultured in 60-mm-diameter plates (6 × 10^5^ cells per plate): CON 242 and COPA 159. After 24 h, cells were transfected using the INTERFERin siRNA transfection reagent (Poly Plus Transfection, New York, NY, USA) with 5 nM siRNA, according to manufacturer's instruction. The following siRNAs were obtained from Dharmacon (Chicago, IL, USA): TDGF1, ON-TARGETplus SMART pool siRNA (anti-CR-1 siRNA) (5′-GCUGGGCCAUCAGGAAUUU-3′, 5′-CAAAGCUACUAUUAAUCGA-3′, 5′-UCAUGGCCAUUUCUAAAGU-3′, 5′-CCGCUUCUCUUACAGUGUG-3′) and siRNA Negative Control Pool. After 24 and 48 h of incubation at 37 °C, invasion assays with siRNA-transfected cells were performed as above described. For the experiments of cell proliferation in the presence of siRNAs, CON 242 and COPA 159 cells were seeded in 48-well cell culture plates. After 24 h, cells were transfected with the anti-CR-1 and the negative control siRNA. Cell growth was measured at the indicated time points with an automated Z1 Coulter Counter (Beckman Coulter, Milan, Italy).

### Flow cytometric analysis

Evaluation of CR-1 expression in CON 242, COPA 159, ROS 184 melanoma cell lines was performed by a direct intracellular immunofluorescent assay. Cells (5 × 10^5^) were incubated in Flow Cytometry Fixation Buffer (R&D Systems) at room temperature for 10 min. Then, cells were washed with phosphate-buffered saline (PBS), re-suspended in Flow Cytometry Permeabilization/Wash Buffer I and incubated with a FITC-conjugated mouse monoclonal anti-human CR-1 antibody (R&D Systems) (50 *μ*g ml^−1^) or with a FITC-conjugated isotype-matched control IgG1 (R&D Systems) for 30 min at 4 °C in the dark. Cells were washed and re-suspended in PBS. Fluorescence was evaluated using a FACScan (Becton Dickinson, Mountain View, CA, USA).

### Western blotting

Levels of expression of CR-1, Nodal and ALK4 were assessed by western blot analysis in selected cell lines, using the CR-1 B3.F6.17 antibody (Adkins *et al*, 2003), the anti-Nodal antibody (Epitomics, Burlingame, CA, USA) and the anti-Activin A Receptor type IB (ALK4) antibody (Epitomics). The blots were normalised by using the anti-*α*-tubulin antibody (clone DM1A) (Sigma). Levels of phospho and total Smad-2 and c-Src were measured following treatment with recombinant CR-1 (100 ng ml^−1^) and/or Nodal (400 ng ml^−1^). The following antibodies were used: (a) phospho-Src family (Tyr 416) rabbit polyclonal antibody (Cell Signalling, Beverly, MA, USA); (b) phospho Smad-2 (Ser 465/467) rabbit monoclonal antibody (Cell Signalling); (c) Src mouse monoclonal antibody (Cell Signalling); (d) Smad-2 mouse monoclonal antibody (Cell Signalling).

### Reverse transcriptase PCR and real-time PCR

Total RNA was isolated from melanoma cell lines using the TRI Reagent (Ambion/Invitrogen, Milan, Italy) according to manufacturer's instruction. The RNA was treated with RNase-free DNase I (Promega Italia, Milan, Italy) to remove potential DNA contamination. Complementary DNA synthesis was performed with Superscript II-Reverse Transcriptase (Invitrogen), using 2 *μ*g aliquots of total cellular RNA and random hexamers as primers.

Real-time PCR for quantification of CR-1 mRNA was carried out with a 7900HT ABI PRISM Instrument (Applied Biosystems, Milan, Italy), using the Taqman technology (Applied Biosystems) as previously described (Normanno *et al*, 2004b).

RT–PCR analysis of Nodal, ALK4 and Activin B was performed as previously described (Adkins *et al*, 2003).

### Statistical analysis

Significance was determined using two-tailed Student's *t*-test. *P*-values <0.05 were considered statistically significant.

## Results

### Expression of CR-1 in human primary cutaneous melanoma and in melanoma cell lines

We assessed the expression of CR-1 by immunohistochemistry in 37 human primary cutaneous melanomas. We found that 16 out of 37 (43%) melanoma samples expressed high levels of CR-1 protein (Table 1). Expression of CR-1 was found in both early and late stages of melanoma development (Table 1). Staining was mostly localised in the cytoplasm, although peri-nuclear and membrane staining was occasionally observed (Supplementary Figure 1).

**Figure 1:**
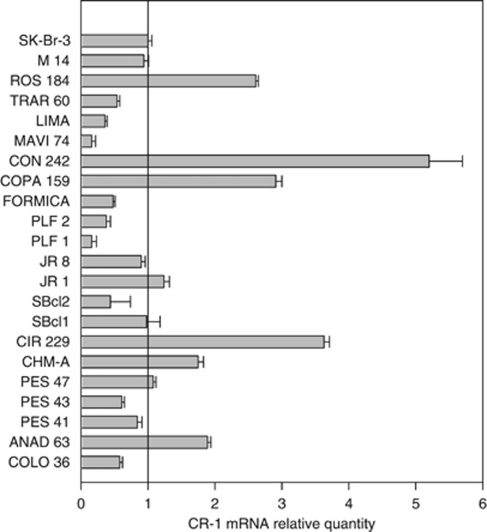
Relative quantity of CR-1 mRNA in a panel of human melanoma cell lines as assessed by real-time PCR. Gene levels were normalised by using GAPDH. The SK-Br-3 cell line was used as a calibrator (relative quantity=1).

To characterise the role of CR-1 in human melanoma, we screened 21 human melanoma cell lines for expression of CR-1 transcripts by real-time PCR and using as calibrator the SK-Br-3 breast cancer cell line that shows relatively low levels of CR-1 mRNA (Normanno *et al*, 2004b). We found that 12 out of 21 cell lines expressed levels of CR-1 mRNA ranging between 0.84 and 5.2 as compared with SK-Br-3 cells (Figure 1). Relative levels of CR-1 mRNA ⩽0.7 were found in 9 out of 21 cell lines. Previous findings in different carcinoma types suggested that cell lines with levels of CR-1 mRNA >0.7 as compared with SK-Br-3 cells might express a biologically active CR-1 protein (Normanno *et al*, 2004b).

**Figure 2:**
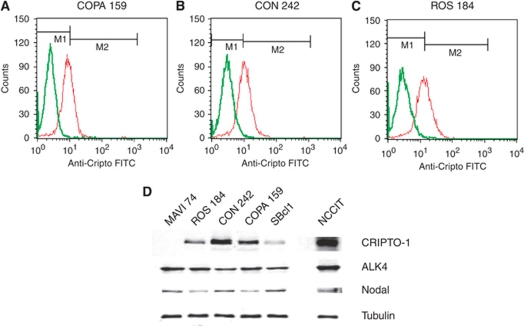
(**A**, **B** and **C**) Cytofluorimetric analysis of CR-1 protein expression in human melanoma cell lines: the green line corresponds to an isotype-matched control IgG1 antibody and the red line to the anti-CR-1 antibody. (**D**) Western blot analysis for CR-1, ALK-4 and Nodal expression in melanoma cells: the testicular cell line NCCIT, that express high levels of CR-1 protein was used as positive control. The blots were normalised with an anti-*α*-tubulin antibody.

Expression of CR-1 protein was assessed by flow cytometric analysis in the human melanoma cell lines ROS 184, CON 242 and COPA 159, which show intermediate to high levels of CR-1 mRNA (Figure 2A–C). In agreement with the real-time PCR data, western blot analysis confirmed expression of intermediate to high levels of CR-1 protein in ROS 184, CON 242 and COPA 159 cells, of low levels in the SBcl1 cell line and no expression in MAVI 74 cells (Figure 2D).

### Expression and activation of CR-1-associated signalling proteins in melanoma cells

In order to assess the expression of factors involved in CR-1-mediated signalling, we examined the expression of Nodal, ALK4 and Activin B mRNAs in melanoma cell lines by RT–PCR. In agreement with previous findings (Topczewska *et al*, 2006), we found expression of specific transcripts for Nodal in 11 out of 15 (73%) analysed cell lines. ALK4 mRNA was expressed in 15 out of 15 (100%) melanoma cell lines, whereas no expression of Activin B mRNA was found in melanoma cells (Supplementary Table 1). We next analysed the expression of Nodal and ALK4 proteins in CON 242, COPA 159, ROS 184, MAVI 74 and SBcl1 cells by western blot analysis (Figure 2D). We found expression of Nodal and ALK4 in all the analysed cell lines, including CON 242 cells that resulted negative for expression of Nodal mRNA by RT–PCR. This discrepancy could be due to low sensitivity of RT–PCR when Dnase-treated RNA is used as template. These data suggest that CR-1/Nodal/ALK4 signalling might be active in the majority of melanoma cell lines.

**Figure 3:**
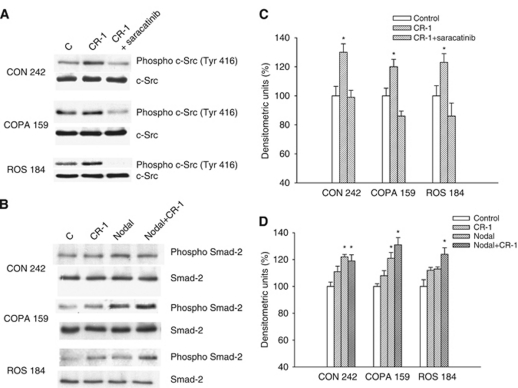
(**A**) Western blot analysis of the total and activated forms of c-Src, following treatment with exogenous recombinant CR-1 or the combination of CR-1 and saracatinib in CON 242, COPA 159 and ROS 184 cells. (**B**) Western blot analysis of the total and activated forms of Smad-2, following treatment with recombinant Nodal, CR-1 and the combination of Nodal and CR-1 in CON 242, COPA 159 and ROS 184 cells. Results are representative of three independent experiments. (**C** and **D**) Densitometric analysis of the blots shown in (**A** and **B**). ^*^P< 0.05 as compared with untreated control, two-tailed Student's *t*-test.

Next, we determined whether treatment of melanoma cells with recombinant CR-1 induces the activation of c-Src and Nodal/ALK4/Smad-2 pathways (Figure 3A and B). Treatment of CON 242, COPA 159 and ROS 184 cells with recombinant CR-1 protein resulted in a significant increase of c-Src phosphorylation, which was prevented by co-treatment with the specific c-Src inhibitor saracatinib (1 *μ*M) (Figure 3A and C). CRIPTO-1, as a co-receptor for the TGF *β*-related proteins Nodal, binds to a complex composed of type I (ALK4, 5 and 7) and type II (ActRIIB) Activin-like kinase receptors, which phosphorylates the downstream transcriptional coactivators Smad-2 and Smad-3 (Bianco *et al*, 2005; Strizzi *et al*, 2005; Gray *et al*, 2006). Therefore, we analysed the effects of CR-1 and/or Nodal on the activation of the Nodal/ALK4/Smad-2 pathway in melanoma cells (Figure 3B and D). As expected, Nodal was able to induce Smad-2 activation in the three melanoma cell lines, and its activity was slightly increased by co-treatment with CR-1. Although statistically significant, the effects of exogenous CR-1 on the activation of Smad signalling were weaker as compared with c-Src.

### Role of CR-1 in melanoma invasiveness

**Figure 4:**
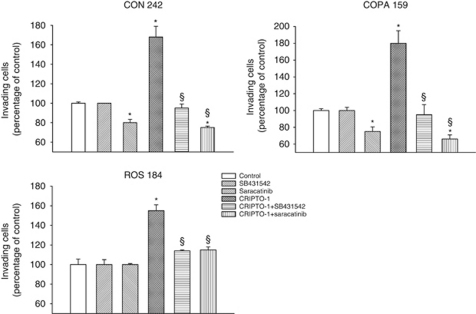
Effects of treatment with CR-1 in presence or absence of the ALK4/5/7 inhibitor SB-431542 or the c-Src inhibitor saracatinib on invasiveness of CON 242, COPA 159 and ROS 184 melanoma cell lines, as determined by using a Boyden chamber-based colorimetric assay. The results are expressed as percentage of control untreated cells. For CON 242 control OD was 0.850 nm, for COPA 159 0.350 nm and for ROS 184 0.656 nm. ^*^P< 0.05 as compared with untreated control; ^§^*P*< 0.05 when the combinations of CR-1 and SB-431542 or saracatinib were compared with CR-1 treated sample, two-tailed Student's *t*-test.

**Figure 5:**
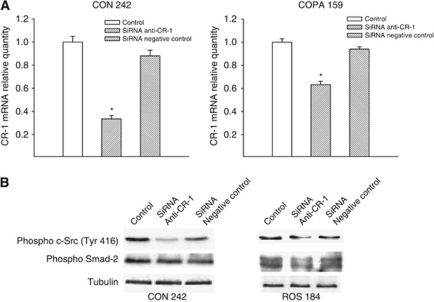
Effects of CR-1 siRNAs on CR-1 expression and intracellular signalling. (**A**) CR-1 mRNA levels were measured in CON 242 and COPA 159 cell lines 24 h after transfection by using real-time PCR. ^*^P<0.05 using two-tailed Student's *t*-test. (**B**) Activation of c-Src and Smad-2 in CON 242 and in ROS 184 cells 24 h after transfection with anti-CR-1 or negative control siRNAs was assessed by western blot analysis. The blots were normalised with an anti-*α*-tubulin antibody. Results are representative of three independent experiments.

Evidence suggests that CR-1 might have a role in the invasiveness of tumour cells. In agreement with this hypothesis, we found that an exogenous recombinant human CR-1 protein (200 ng ml^−1^) induced a significant increase in the invasive ability of CON 242, COPA 159 and ROS 184 melanoma cell lines (Figure 4). Treatment of melanoma cells with saracatinib (0.5 *μ*M) or the ALK4/5/7 inhibitor SB-431542 (10 *μ*M) inhibited CR-1-induced invasion (Figure 4). In CON 242 and COPA 159 cells, saracatinib also affected the basal invasive activity. This phenomenon might be due to the activation of c-Src signalling by endogenous CR-1 or by other factors that activate c-Src. In order to address this phenomenon, we analysed the effects of silencing endogenous CR-1 by transfection of melanoma cell lines with a pool of four CR-1 siRNAs. We observed a significant reduction in the levels of CR-1 mRNA in CON 242 and COPA 159 transfected with the anti-CR-1 siRNA as compared with untreated cells and cells treated with a negative control siRNA (Figure 5A). In agreement with these findings, transfection of COPA 159 and CON 242 cell lines with anti-CR-1 siRNAs produced a significant reduction of CR-1 protein expression after 48 h of treatment, as assessed by flow cytometry (data not shown). We next analysed the effects of anti-CR-1 siRNA on the activation of the c-Src and Nodal/ALK4/Smad-2 pathways. Treatment of CON 242 with anti-CR-1 siRNAs resulted in a marked reduction of phospho-Src, whereas it produced a slight reduction of phosho-Smad-2 (Figure 5B).

In ROS 184 cells that express lower levels of CR-1, treatment with siRNAs resulted in a similar reduction of CR-1 expression (data not shown). However, silencing of CR-1 in this latter cell line produced slight effects on the activation of c-Src and Smad-2 (Figure 5B).

**Figure 6:**
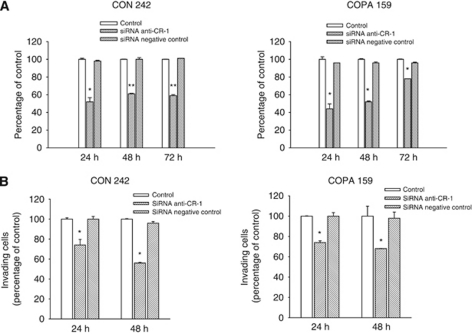
Effects of CR-1 silencing on the growth and invasion of melanoma cell lines. (**A**) The effects of anti-CR-1 or control siRNAs on the proliferation of CON 242 and COPA 159 cell lines were determined at the indicated time points with an automated cell counter. ^*^P<0.05, ^**^P<0.0001, two-tailed Student's *t* test. (**B**) Invasion of melanoma cells was evaluated 24 and 48 h after transfection by using a Boyden chamber-based colorimetric assay. ^*^P<0.05, two-tailed Student's *t*-test.

We next investigated whether the silencing of endogenous CR-1 affects the proliferation and invasive ability of melanoma cells that express high levels of CR-1. In CON 242 and COPA 159 cells, a significant reduction on cell growth was observed following transfection with anti-CR-1 siRNA (Figure 6A). In addition, a time-dependent inhibition of the invasive ability of melanoma cells was observed after silencing of endogenous CR-1 mRNA (Figure 6B). In melanoma cells treated with negative siRNA control, no effects on cell invasion were observed.

Treatment with anti-CR-1 siRNAs slightly reduced the proliferation and the invasion of ROS 184 cells (<20% reduction) (data not shown). These findings are consistent with the observation that saracatinib did not affect the basal levels of invasion of ROS 184 cells.

### Effects of saracatinib on melanoma cell proliferation

**Figure 7:**
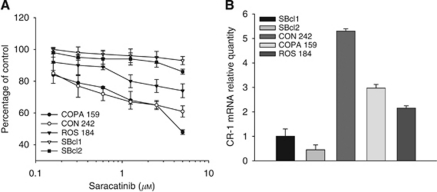
(**A**) Effects of treatment with saracatinib on the proliferation of CON 242, COPA 159, ROS 184, SBcl1 and SBcl2 cell lines. (**B**) Relative quantification of CR-1 mRNA in melanoma cell lines.

We assessed the effects of saracatinib on the proliferation of a small panel of melanoma cell lines including CON 242, COPA 159, ROS 184, SBcl1 and SBcl2 cells. Two cell lines (SBcl1 and SBcl2) were resistant to the growth inhibitory effect of the drug (maximum 10% growth inhibition at the highest concentration), ROS 184 cells were slightly inhibited (25% growth inhibition), whereas CON 242 and COPA 159 cells showed a moderate sensitivity (40–50% growth inhibition) (Figure 7A). At the concentration of 0.5 *μ*M, the effects of saracatinib on the proliferation of the cell lines with the highest levels of expression of CR-1 (CON 242 and COPA 159) were statistically significant (*P*<0.05, two-tailed Student's *t*-test), whereas the difference for the cell lines with low or intermediate levels of expression of CR-1 were not significant at this drug concentration. Interestingly, a strong correlation was found between the expression of CR-1 mRNA and the response to saracatinib in our panel of melanoma cell lines (Figure 7B).

## Discussion

This paper is the first to demonstrate that CR-1 is expressed in ∼50% of human primary cutaneous melanomas and melanoma cell lines. Expression of CR-1 has been recently described in primary uveal melanomas and uveal melanoma cell lines (Mallikarjuna *et al*, 2007). Interestingly, in uveal melanoma the expression of CR-1 was significantly higher in cases with extra-scleral extension/liver metastasis as compared with melanomas with no extension. In our cohort of patients, expression of CR-1 was found both in early and late-stage cutaneous melanoma.

Our paper is also the first to formally demonstrate that CR-1 can function as an autocrine and/or paracrine growth factor in melanoma cells, in which CR-1 is able to activate both Nodal and Src signalling. In this regard, recent findings have demonstrated that Nodal, an embryonic morphogen belonging to the TGF*β* superfamily, may have an important role in melanoma pathogenesis (Topczewska *et al*, 2006; Postovit *et al*, 2008). Nodal was found to be expressed in metastatic melanoma, and inhibition of Nodal signalling significantly reduced the ability of melanoma cells to invade, to form colonies and to reproduce tumours in immunocompromised mice (Topczewska *et al*, 2006). CRIPTO-1 interacts with Nodal signalling at different levels. Biochemical evidence has demonstrated a direct interaction between mouse Cripto and ALK4 that facilitates the binding of Nodal to the ALK4/ActRIIB receptor complex and the activation of Smad-2 (Yeo and Whitman, 2001; Bianco *et al*, 2002). Cripto can also directly interact with the serine threonine kinase ALK7 receptor enhancing its ability to respond to Nodal (Reissmann *et al*, 2001). In this regard, we found that the majority of melanoma cell lines that express CR-1 have also transcripts for Nodal and ALK4, suggesting that a Nodal/CR-1/ALK4 pathway might be active in melanoma. More recently, Cripto was found to interact with Nodal during endocytosis and secretion (Blanchet *et al*, 2008a, 2008b). Binding of Cripto to Nodal during endocytosis leads to enrichment of Nodal at the limiting membrane of early endosomes, where it can easily interact with cytoplasmic effectors (Blanchet *et al*, 2008b). Cripto-mediated endocytosis of Nodal thus may primarily serve to prolong Nodal signalling. Furthermore, it has been demonstrated that Cripto can bind to the Nodal precursor protein during exocytosis thereby enabling the assembly of a complex with the convertases Furin and PACE4 involved in the processing of Nodal (Blanchet *et al*, 2008a). It appears, therefore, that both extracellular and intracellular pools of Cripto have important roles during Nodal processing, secretion and signalling. In this regard, our findings suggest that expression of CR-1 might significantly enhance the metastatic potential of melanoma cells through the activation of the Nodal/ALK/Smad pathway. Although treatment with recombinant CR-1 and Nodal only slightly enhanced the activation of Smad-2 in melanoma cells, blockade of ALK activation with a specific inhibitor was able to significantly reduce the ability of CR-1 to stimulate melanoma cell invasion, suggesting that Nodal/Smad-2 signalling has an important role in CR-1-induced invasiveness.

CRIPTO-1 can also bind to Glypican-1, a membrane-associated heparan sulphate proteoglycan that functions as a co-receptor for several different growth factors (i.e., Wnts, FGF, HB-EGF) (Bianco *et al*, 2003). Binding of CR-1 to Glypican-1 activates the cytoplasmic tyrosine kinase c-Src, triggering the activation of MAPK and AKT signalling pathways independently of Nodal and ALK4 (Bianco *et al*, 2003). Src family kinases regulates key pathways in metastasis including cell adhesion, invasion and motility, and regulate proliferation and survival of tumour cells in response to activation of growth factor receptors (Homsi *et al*, 2007). Evidence suggests that src signalling is implicated in melanoma progression (Niu *et al*, 2002; Homsi *et al*, 2009; Putnam *et al*, 2009). Expression of both c-Src and Yes has been reported to be elevated in melanoma cells as compared with normal melanocytes (Barnekow *et al*, 1987; Loganzo *et al*, 1993). Approximately 50% of melanomas were found to express phosphorylated c-Src (Homsi *et al*, 2009). In addition, c-Src becomes activated at the heterotypic contact between the transmigrating melanoma cell and the neighbouring endothelial cells during tumour cell transendothelial migration, a critical step in cancer metastasis (Qi *et al*, 2006). Finally, different reports have demonstrated that treatment of melanoma cells with the c-Src inhibitor dasatinib results in significant reduction of motility and invasion (Buettner *et al*, 2008; Eustace *et al*, 2008). In this regard, our findings confirm that Src signalling is an important mediator of melanoma cell invasiveness. Indeed, treatment with the c-Src inhibitor saracatinib produced a significant reduction of melanoma cell invasion. In addition, we found that recombinant CR-1 was able to induce a significant increase in the levels of activation of c-Src in melanoma cells. In agreement with these findings, in melanoma cell lines saracatinib completely abolished the ability of exogenous CR-1 to stimulate invasion of these cells. Saracatinib but not SB-431542 also inhibited the basal invasion of serum-starved melanoma cells, suggesting that in these growth conditions c-Src signalling is driving the invasion of melanoma cells. The observation that CR-1 siRNAs reduced the invasive ability of melanoma cells and the activation of c-Src supports the hypothesis that endogenous CR-1 might induce the growth and invasion of melanoma cells through activation of c-Src. However, this phenomenon might be restricted to the cell lines that express high levels of CR-1 protein, as saracatinib had no effects on the basal levels of invasion of ROS 184 that express intermediate levels of CR-1 protein (Figure 2). Interestingly, transfection with CR-1 siRNA had no significant effect on the activation of c-Src in ROS 184 cells (Figure 5B). Endogenous CR-1 seems to regulate mainly c-Src activation in melanoma cells. No data are available to sustain that exogenous and endogenous CR-1 might have different effects on cell signalling. The difference that we found might be simply related to the levels of CR-1 that are available at cellular level to activate intracellular signalling pathways. In addition, CR-1 is not able to activate directly ALK receptors, but rather acts as a co-receptor for Nodal, which might represent a limiting factor for the activation of ALK/Smad signalling.

Previous reports have shown that dasatinib has no major effects on the proliferation of melanoma cells, with a maximum 40–50% growth inhibition observed in the most sensitive cell lines for concentrations of the drug up to 5 *μ*M (Buettner *et al*, 2008; Eustace *et al*, 2008; Homsi *et al*, 2009). Saracatinib showed a growth inhibitory activity on melanoma cell lines that was comparable to dasatinib, with two cell lines showing resistance to this drug, one cell line having a marginal sensitivity and two cell lines showing moderate sensitivity. Importantly, a close correlation was found in this small panel of melanoma cell lines between the levels of expression of CR-1 and sensitivity to saracatinib.

In conclusion, this study demonstrates that a significant fraction of melanoma expresses CR-1, and that this growth factor is involved in the invasion and proliferation of melanoma cells. Our data also suggest that the expression of CR-1 in melanoma cells might represent a marker of Src activation and, therefore, of sensitivity to c-Src inhibitors such as saracatinib. Further studies are in progress to assess whether CR-1-mediated signalling might represent a novel target for therapeutic intervention in melanoma.

## Figures and Tables

**Table 1:** Immunohistochemical analysis of CR-1 expression in human melanomas

**Lesion**	**CR-1 positive**	**%**
Early-stage melanoma	8/16	50
Late-stage melanoma	8/21	38

Abbreviation: CR-1=CRIPTO-1.
