# Supplementary material for: Expression and functional role of CRIPTO-1 in cutaneous melanoma
Source: Br J Cancer. 2011 Aug 23;105(7):1030–8. doi: 10.1038/bjc.2011.324 (PMC3185940; doi:10.1038/bjc.2011.324)
Supplement: Supplementary Figure 1 [file bjc2011324x1.ppt]

## Slide 1
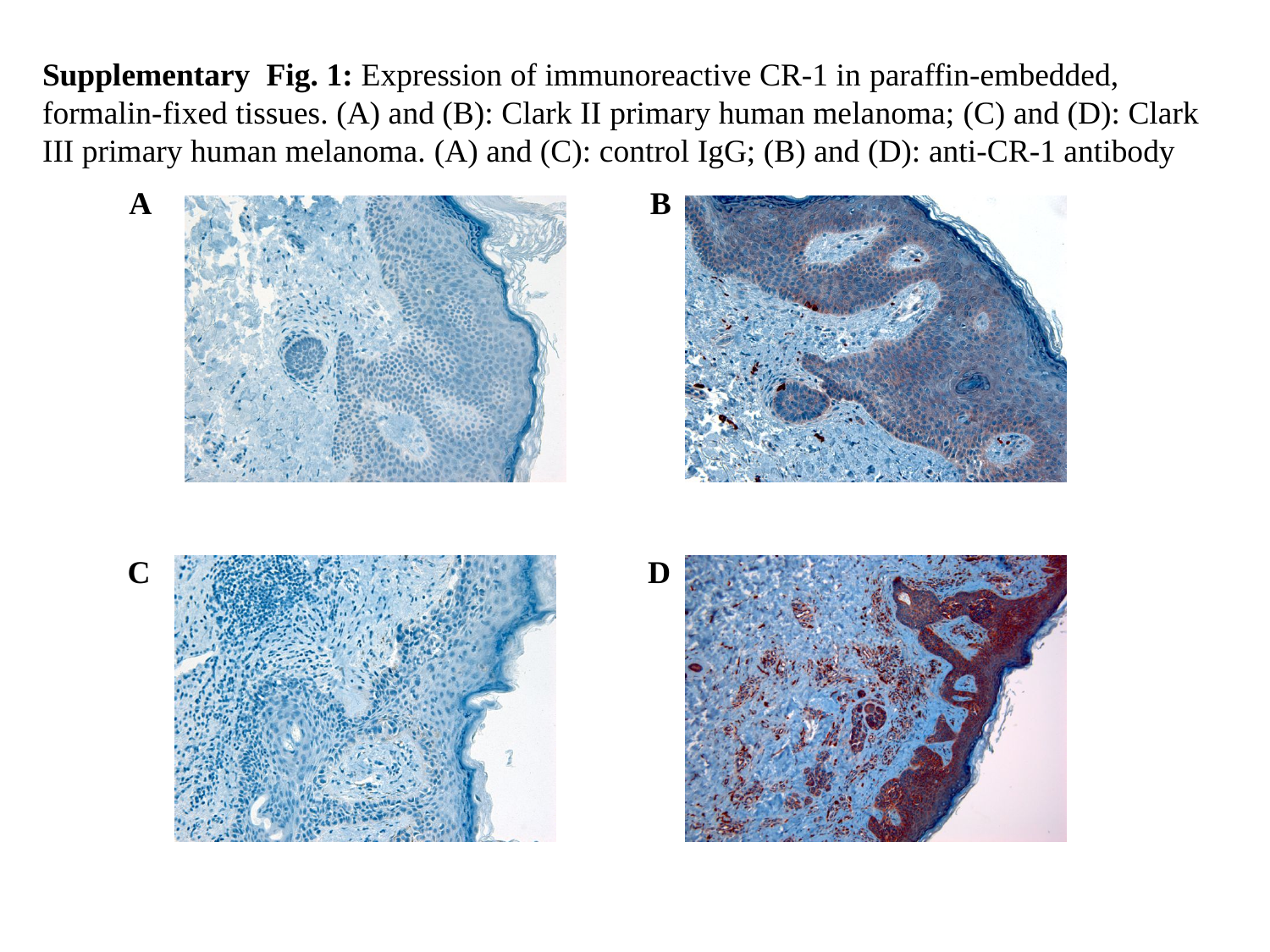

Supplementary Fig. 1: Expression of immunoreactive CR-1 in paraffin-embedded, formalin-fixed tissues. (A) and (B): Clark II primary human melanoma; (C) and (D): Clark III primary human melanoma. (A) and (C): control IgG; (B) and (D): anti-CR-1 antibody
A
B
C
D
