# Supplementary material for: Expression and functional role of CRIPTO-1 in cutaneous melanoma
Source: Br J Cancer. 2011 Aug 23;105(7):1030–8. doi: 10.1038/bjc.2011.324 (PMC3185940; doi:10.1038/bjc.2011.324)
Supplement: Supplementary Table 1 [file bjc2011324x2.doc]

**Supplementary Table 1.** Expression of Nodal and ALK-4 mRNA in melanoma cell lines

|  | Nodal | Alk-4 | Activin B |
| --- | --- | --- | --- |
| M 14 | + | + | - |
| ROS 184 | + | + | - |
| TRAR 60 | - | + | - |
| LIMA | + | + | - |
| MAVI 74 | + | +/- | - |
| CON 242 | - | + | - |
| COPA 159 | + | + | - |
| PLF 2 | + | + | - |
| JR1 | + | + | - |
| SBcl 2 | + | + | - |
| SBcl 1 | + | + | - |
| CIR 229 | - | +/- | - |
| CHM-A | - | + | - |
| PES 43 | + | + | - |
| ANAD 63 | + | + | - |

+/- indicates a dim band
